# Cyanobacteria and the Great Oxidation Event: evidence from genes and fossils

**DOI:** 10.1111/pala.12178

**Published:** 2015-06-23

**Authors:** Bettina E. Schirrmeister, Muriel Gugger, Philip C. J. Donoghue

**Affiliations:** ^1^ School of Earth SciencesUniversity of BristolLife Science Building24 Tyndall AvenueBristolBS8 1TQUK; ^2^ Institut PasteurCollection des Cyanobactéries75724Paris Cedex 15France

**Keywords:** early life, divergence time estimation, genomics, atmosphere, major transition

## Abstract

Cyanobacteria are among the most ancient of evolutionary lineages, oxygenic photosynthesizers that may have originated before 3.0 Ga, as evidenced by free oxygen levels. Throughout the Precambrian, cyanobacteria were one of the most important drivers of biological innovations, strongly impacting early Earth's environments. At the end of the Archean Eon, they were responsible for the rapid oxygenation of Earth's atmosphere during an episode referred to as the Great Oxidation Event (GOE). However, little is known about the origin and diversity of early cyanobacterial taxa, due to: (1) the scarceness of Precambrian fossil deposits; (2) limited characteristics for the identification of taxa; and (3) the poor preservation of ancient microfossils. Previous studies based on 16S rRNA have suggested that the origin of multicellularity within cyanobacteria might have been associated with the GOE. However, single‐gene analyses have limitations, particularly for deep branches. We reconstructed the evolutionary history of cyanobacteria using genome scale data and re‐evaluated the Precambrian fossil record to get more precise calibrations for a relaxed clock analysis. For the phylogenomic reconstructions, we identified 756 conserved gene sequences in 65 cyanobacterial taxa, of which eight genomes have been sequenced in this study. Character state reconstructions based on maximum likelihood and Bayesian phylogenetic inference confirm previous findings, of an ancient multicellular cyanobacterial lineage ancestral to the majority of modern cyanobacteria. Relaxed clock analyses provide firm support for an origin of cyanobacteria in the Archean and a transition to multicellularity before the GOE. It is likely that multicellularity had a greater impact on cyanobacterial fitness and thus abundance, than previously assumed. Multicellularity, as a major evolutionary innovation, forming a novel unit for selection to act upon, may have served to overcome evolutionary constraints and enabled diversification of the variety of morphotypes seen in cyanobacteria today.

Cyanobacteria, oxyphototrophic eubacteria, are among the most important and influential organisms in the evolution of an aerobic Earth in the Precambrian (Rippka *et al*. 1979; Castenholz 2001). Cyanobacteria are the only organisms in which oxygenic photosynthesis has evolved. A characteristic obtained by other organisms, such as plants and algae, through endosymbiosis with cyanobacteria, resulting in the formation of chloroplasts (Sagan 1967). During the early Proterozoic, 2.5–2.3 Ga, atmospheric oxygen levels accumulated rapidly during an episode coined the Great Oxidation Event (GOE; Bekker *et al*. 2004; Anbar *et al*. 2007) as a result of cyanobacterial activity leading to great changes to the Earth system (Van Kranendonk *et al*. 2012), subsequently allowing for the evolutionary emergence of complex life as we know it.

The autotrophic lifestyle of cyanobacteria enabled them to conquer a variety of habitats including marine, limnic and soil environments, ranging over a wide scale of temperatures, from arctic regions to hot springs. In addition to their ecological variety, cyanobacteria exhibit impressive morphological diversity, particularly for prokaryotes. On this basis, cyanobacteria have been classified and divided into five subsections (Rippka *et al*. 1979; Castenholz 2001). Although not confirmed by molecular data, this taxonomic system still proves useful for cyanobacterial classification. Subsections I and II comprise unicellular cyanobacteria; subsection I cyanobacteria (e.g. *Synechococcus*,* Synechocystis*) reproduce via binary fission or budding (e.g. *Chamaesiphon*), whereas subsection II cyanobacteria reproduce via a rapid division of a vegetative ‘mother’ cell into smaller, spherical cells termed ‘baeocysts’ (Rippka *et al*. 1979). Subsections III to V include filamentous cyanobacteria with varying levels of multicellular complexity. Reproduction in these taxa occurs via filament (trichome) breakage and the formation of short motile filaments termed ‘hormogonia’. While subsection III cyanobacteria grow uniseriate (one cell width), unidirectional filaments in one plane, bacteria in subsections IV and V have the ability to form differentiated cells, such as metabolically specialized heterocysts for nitrogen fixation and resting cells, so called akinetes (Flores and Herrero 2010). Taxa belonging to subsection V are the only cyanobacteria that grow multiseriate filaments (varying cell width) and in several planes exhibit ‘true’ branching, not to be confused with ‘false’ branching (Rippka *et al*. 1979; Gugger and Hoffmann 2004).

This variety in shapes allows for a fairly reliable identification of cyanobacterial relatives in the fossil record compared to other prokaryotes. Fossil cyanobacteria from almost all subsections have been found in Mesoproterozoic deposits (Schopf and Blacic 1971; Horodyski and Donaldson 1980; Butterfield 2000). Cyanobacteria belonging to subsections I and III have been identified in the early Proterozoic including the 1.8 Ga Duck Creek Dolomite in Australia (Knoll *et al*. 1988) and 1.9 Ga Belcher Supergroup in Canada (Hofmann 1976). Furthermore, 2.0 Ga fossilized resting cells (present in subsections IV and V), termed ‘*Archaeoellipsoides*’, resemble akinetes of modern subsection IV cyanobacterial genus *Anabaena* (Amard and Bertrand‐Sarfati 1997). The appearance of cyanobacterial fossils attributed to subsections IV and V cyanobacteria occurs after the GOE, supporting the hypothesis that heterocyst formation evolved after the increase in atmospheric oxygen to allow for nitrogen fixation, spatially separated from photosynthesis (Tomitani *et al*. 2006). The diversity of cyanobacterial taxa soon after the GOE indicates an origin of cyanobacterial ancestors some time in the Archean. Yet, after the debate around 3.5‐Gyr‐old cyanobacterial‐like fossils (Schopf 1993; Brasier *et al*. 2002; Schopf and Kudryavtsev 2009) and the misidentification of 2.7‐Gyr‐old cyanobacterial biomarkers (Brocks *et al*. 1999; Rasmussen *et al*. 2008), the existence of Archean cyanobacteria has been questioned. Although many multicellular fossil remains from the Archean and Proterozoic (Altermann and Schopf 1995; Schopf 2006) have been interpreted as cyanobacteria, the overall fossil record is patchy at best and provides only glimpses into the history of these early prokaryotes. However, combining molecular phylogenomics with existing palaeontological data to approximate the evolution of prokaryotes in the Precambrian allows us to evaluate the co‐evolution of early life and Earth and helps us to resolve questions regarding the origin of complex life today.

Current competing hypotheses that attempt to explain the origin of cyanobacteria include: (1) an origin of cyanobacteria immediately followed by the GOE (Kirsch‐vink and Kopp 2008); and (2) a much earlier origin of cyanobacteria, based on the, at least temporary, accumulation of oxygen in Earth's oceans and atmosphere, around *c. *2.8–3.0 Ga (Crowe *et al*. 2013; Lyons *et al*. 2014). These hypotheses, however, ignore the possibility of evolutionary innovations that could have influenced fitness and, hence, abundance of cyanobacteria. Could evolutionary innovations in cyanobacteria have triggered the rapid oxygenation during the GOE? Previous studies have hypothesized that the origin of multicellularity in cyanobacteria might be causally associated with the GOE (Schirrmeister *et al*. 2013). In heterotrophic prokaryotes, metabolic rates have been found to increase superlinearly with body mass (DeLong *et al*. 2010). The transition to multicellularity with its increase in overall size could have had a similar effect in cyanobacteria.

Unfortunately, attempts to resolve the early evolutionary history of life using molecular phylogenetic approaches has reached contradictory conclusions due to a lack of data and weaknesses in methodology (Schirrmeister *et al*. 2013; Shih and Matzke 2013). Difficulties in obtaining accurate age estimates for such deep speciation events may include: (1) the lack of evolutionary information retained in molecular data over such long timescales; (2) weaknesses in the phylogenetic model to correctly infer evolutionary processes that took place over such long time periods; and (3) the paucity of calibration points for divergence time estimation. Further difficulties of calibrations involve: (1) the potential for their misassignment to clades; and (2) the likelihood that the age of the calibrating fossils is a poor approximation of their clade age. Although the limiting factor on precision in divergence time studies is invariably the uncertainty associated with the fossil calibrations (dos Reis and Yang 2013), in terms of the degree to which fossil minima approximate the true time of divergence, much of the uncertainty associated with phylogeny and divergence time estimation can be diminished by employing genome scale data (dos Reis *et al*. 2012).

In this study we aim to: (1) provide more reliable age estimates for the evolutionary history of Cyanobacteria, based on a large data set involving 756 highly conserved proteins (197 761 amino acids) and (2) compare Precambrian fossil findings to modern cyanobacteria in order to identify improved calibration priors. Sampling of cyanobacterial taxa for phylogenomic analyses was based on a previously published 1220 taxa‐rich phylogenetic tree (Schirrmeister *et al*. 2011a). Adding to an existing set of genomes recently obtained in the frame of diversity‐driven representation of the cyanobacterial phylum (Dagan *et al*. 2013; Shih *et al*. 2013), we sequenced eight additional cyanobacterial genomes. We tested established hypotheses of relationships among these species through maximum likelihood phylogenetic analyses. The resulting tree formed the basis for subsequent analyses that aimed to: (1) reconstruct multicellular character states; and (2) estimate divergence times using a relaxed molecular clock. Phylogenomic results show increased tree support compared to single‐gene analyses and confirm previous suggestions of early multicellularity in cyanobacteria (Schirrmeister *et al*. 2013).

## Material and methods

### Taxon sampling

Cyanobacterial genomes included in the phylogenetic studies were chosen so that: (1) representatives of all five subsections were used; and (2) the entire molecular diversity of this phylum was covered (Schirrmeister *et al*. 2011a, *b*). Previously oversampled groups for genomic analyses, such as marine picocyanobacteria (*Synechococcus* and *Prochlorococcus*), were down‐sampled, so that the inferred tree depicts known phylogenetic disparity of the phylum as recovered 16S rRNA data. The phylogenetic analyses included genomic data of 27 unicellular (section I and II) and 38 multicellular (section III to V) taxa (Table S1). Following the comparison of 16S rRNA phylogenies and phylogenomic studies (Schirrmeister *et al*. 2011b; Shih *et al*. 2013), eight additional cyanobacterial taxa were sampled for genome sequencing to investigate the early split of multicellular cyanobacterial taxa and to evaluate the suggested polyphyly of subsection II cyanobacteria. The strains represented the unicellular *Synechocystis* sp. PCC 9635, the unicellular and baeocystous *Chroococcidiopsis* sp. PCC 8201 and five multicellular strains belonging to the filamentous cyanobacteria without cellular differentiation: (1) *Geitlerinema* sp. PCC 8501; (2) *Leptolyngbya* sp. PCC 73110; (3) *Limnothrix redekei* PCC 9416; (4) *Pseudanabaena* sp. PCC 7704 and PCC 7904; and (5) *Symploca* sp. PCC 8002).

### Genome reconstructions

Genomic DNA was extracted using the NucleoBond^®^AXG provided by Macherey‐Nagel (www.mn-net.com) as previously performed for the CyanoGEBA (Shih *et al*. 2013). Sequencing of eight cyanobacterial genomes was conducted at the Transcriptomics Facility of the University of Bristol using an Illumina Genome Analyzer IIx and Illumina HiSeq 2500. Paired‐end reads were 100 bp in length and coverage for all genomes was >100× coverage. From the resultant sequences, low‐quality reads, sequencing adapters and primers were removed using the adapter cleaning program Fastq‐mcf (Aronesty 2013). An initial *de novo* assembly of newly sequenced cyanobacterial genomes was conducted applying Velvet v1.2.10 software (Zerbino and Birney 2008). Optimal K‐mer lengths for a sensitive, yet specific assemblage, were determined separately for each genome based on highest N50 values and maximum contig length. The genome assembly was further improved aligning contigs on an artificially reconstructed reference genome in ABACAS v1.3.1 (Assefa *et al*. 2009), which were reconstructed based on the Velvet assembler using EMBOSS‐union software (Rice and Breasby 2011). Gaps in draft assemblies were reduced, and overall genome completeness was further improved using the Iterative Mapping and Assembly for Gap Elimination (IMAGE) software (Tsai *et al*. 2010). Both ABACES and IMAGE were applied as part of the Post Assembly Genome Improvement Toolkit (PAGIT; Swain *et al*. 2012). Annotation of sequences was conducted via a rapid prokaryotic sequence annotation algorithm implemented in Prokka v1.5.2 (Seemann 2014). For further analyses, a database was constructed including the genomes of 57 cyanobacterial taxa from GenBank, chosen to best represent phylogenetic disparity. Highly conserved protein sequences were identified applying BlastP for protein–protein similarity searches as part of the ‘Basic Local Alignment Tool’ algorithm Blast+ (Camacho *et al*. 2009).

### Phylogenomic analyses using a supermatrix

**Figure 1:**
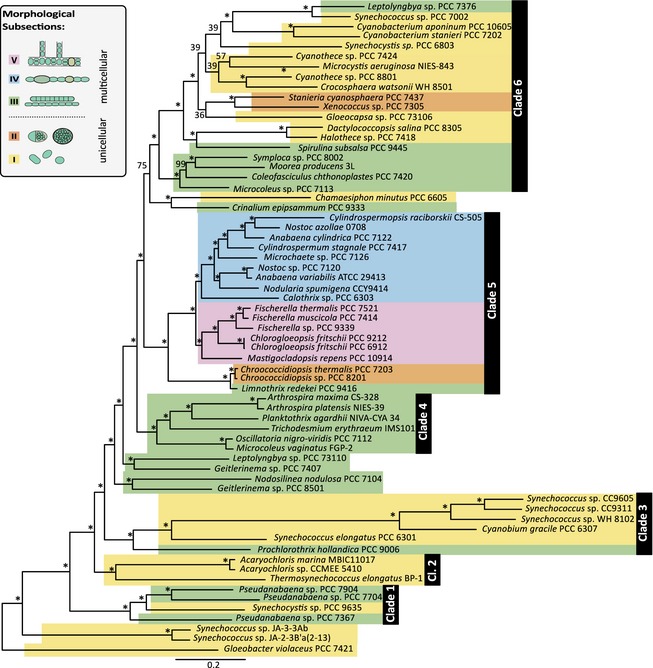
Phylogenomic maximum likelihood tree. Phylogeny of 65 cyanobacterial taxa based on a supermatrix comprised of 756 concatenated protein sequences (197 761 amino acid sites). Maximum likelihood bootstrap support for clades is indicated at respective branches. Stars indicate 100% support calculated from 1000 bootstrap resamplings. Cyanobacterial taxa are colour‐coded. Unicellular taxa belonging to morphological subsections I and II are displayed in yellow and orange, respectively, whereas multicellular cyanobacterial taxa belonging to subsections III, IV and V are shown in green, blue and pink, respectively. The majority of branches in this phylogeny are well supported. Six distinct clades could be reconstructed with full support. Differentiated cyanobacteria belonging to subsections IV and V are the only groups where morphological and genomic data congruently suggest a monophyletic origin.

Multiple sequence alignments including all 65 cyanobacterial taxa (Table S1) were reconstructed for each of the 756 identified proteins (Schirrmeister *et al*. 2015, appendix S1) using Mafft‐7.058 (Katoh and Standley 2013). Sites where gaps were present in 75% of the taxa were removed using Phyutility (Smith and Dunn 2008). The multiple sequence alignments of all 756 proteins were concatenated, and further analyses were conducted on the resultant 197 761 amino acid‐rich supermatrix (Schirrmeister *et al*. 2015, appendix S2). The best‐fitting model to approximate amino acid evolution was identified applying the software ProtTest 3.2 (Darriba *et al*. 2011). The LG + Γ model (Le and Gascuel 2008) was identified as the best‐fitting model. Phylogenetic trees based on a maximum likelihood algorithm were reconstructed using the parallelized version RAxML‐7.2.8‐pthreads for multicore processor architectures (Stamatakis *et al*. 2005) run on the High Performance Computing machine (Bluecrystal) at the Advanced Computing Research Centre of the University of Bristol. Of 10 maximum likelihood trees, the one with the highest likelihood score was chosen (Fig. 1; Schirrmeister *et al*. 2015, appendix S3). Bootstrap analyses for the concatenated data set were run 1000 times.

### Phylogenetic reconstructions based on 16S and 23S rRNA

16S and 23S rRNA sequences of the 65 cyanobacterial taxa were aligned using Mafft‐7.058 (Katoh and Standley 2013) and concatenated. The Akaike Information Criterion, implemented in jModelTest v0.1.1 (78), suggested a general time‐reversible substitution model with gamma‐distributed rate variation among sites (GTR + Γ; Lanave *et al*. 1984) as the most suitable model of sequence evolution. Phylogenetic trees were reconstructed based on maximum likelihood using RAxML‐7.2.8 (Stamatakis *et al*. 2005). Ten maximum likelihood analyses were conducted, of which the one with the best likelihood is presented (Fig. S1; Schirrmeister *et al*. 2015, appendix S3). Phylogenetic support was gathered from 1000 bootstrap resamplings.

### Ancestral character state reconstruction

Ancestral character states were reconstructed using the ‘ace’ function of the ape package (Paradis *et al*. 2004) in R (http://www.r-project.org/.). Ancestral states were reconstructed applying maximum likelihood methods assuming equal and asymmetrical transition rates. q01 describes the transition to multicellularity and q10 the transition to unicellularity.

Character states were reconstructed using continuous‐time Markov models as implemented in BayesTraits v.2 beta (Pagel *et al*. 2004). Markov chain Monte Carlo (MCMC) analysis were run for 1 010 000 iterations, with a default burn‐in of 10 000. Convergence of all parameters was reached at this point. Transition rates q01 and q10 were sampled from a uniform prior distribution with the assumption that any rate within this interval is equally likely. Four analyses were conducted with different ranges for the uniform distribution (Table S2).

### Divergence time estimation and calibration

Divergence times were estimated applying relaxed clocks with lognormal rate distribution, which incorporates lineage‐specific rate heterogeneity (Drummond *et al*. 2006). Previous studies have confirmed: (1) the monophyly of the phylum Cyanobacteria; and (2) *Gloeobacter violaceus* closest to a eubacterial outgroup (Blank and Sanchez‐Baracaldo 2010; Schirrmeister *et al*. 2011b; Shih *et al*. 2013). The position of *G. violaceus* outside a cyanobacterial ingroup is further supported by morphological and genetic differences, such as the lack of thylakoid membranes (Rippka *et al*. 1974) and many missing genes associated with the photosystems (Nakamura *et al*. 2003). Divergence time estimation was conducted using the software MCMCTREE, part of the PAML 4.4b package (Yang 2007), and run on the High Performance Computing machine (Bluecrystal) at the Advanced Computing Research Centre of the University of Bristol. Branch lengths were estimated using the software CODEML, also part of the PAML package. Again, LG + Γ (Le and Gascuel 2008) was selected as the model of evolution. Based on the estimated substitution rates, we used a gamma distribution with a shape parameter of 1 and a scale param‐eter of 9.53, as prior for the overall substitution rates. In a first run without calibration priors, the gradient vector and Hessian matrix were calculated. For each analysis, we ran four Markov chain Monte Carlo analyses for 10 million generations, sampling every 100 generations; 50% were removed as burn‐in. At this point, all parameters had reached convergence. Using divergence time estimation, two hypotheses were investigated with different combinations of three calibrations (Table 1). User‐specified time priors and the ‘effective’ (joint) time prior implemented in the calculation of the posterior ages may differ substantially (Warnock *et al*. 2012). Therefore, we conducted the analyses without sequence data to obtain an estimate of the effective priors and to ensure that these do not differ unreasonably from our specified time priors.

**Table 1:** Priors for the tree calibration

Analysis	Calibration	Distribution	Hypothesis 1		Hypothesis 2	
A, C	Root	Uniform (hb)	2.45	3.85	2.45	3.85
	Section IV and V	Uniform (sb)	1.957	2.32	1.957	2.32
	Node 3	Uniform (sb)	1.957	2.45	2.45	3.33
B, D	Root	Uniform (hb)	2.918	3.85	2.918	3.85
	Section IV and V	Uniform (sb)	1.957	2.32	1.957	2.32
	Node 3	Uniform (sb)	1.957	2.45	2.45	3.33

Hypothesis 1 assumes an origin of multicellularity at node 69 after the GOE. In this case, node 69 is calibrated with a uniform distribution between 2.45 Ga (beginning of the GOE) and 1.957 Ga (first multicellular cyanobacterial fossils). Hypothesis 2 assumes an origin of multicellularity before the GOE; hence, a uniform calibration is used between 3.33 Ga (filamentous fossils that show no evidence for a cyanobacterial relation) and 2.45 Ga (the GOE). The root has been calibrated assuming that cyanobacteria originated some time after the late heavy bombardment (3.85), but (A, C) before the GOE (2.45) or (B, D) before the first accumulation of free oxygen (2.918 Ga). Additionally, the node leading to subsections IV and V cyanobacteria capable of cellular differentiation has been calibrated. Fossil akinetes (1.957 Ga) indicate a presence of this clade at 1.957 Ga. Yet, a differentiation of heterocysts does only show selective advantage at oxygen levels after the GOE (2.32 Ga).

hb (hard bounds): allow for no estimates outside the prior densities during the analyses; sb (soft bounds): age estimates may fall outside the effective priors with a 5% probability.

### Restricting the origin of cyanobacteria (calibration 1)

Due to its affinity to a eubacterial outgroup, *Gloeobacter* was treated as outgroup to the rest of Cyanobacteria. The divergence of *Gloeobacter* lineage from the stem lineage leading to the all other extant cyanobacterial species was assumed to have occurred some time between 3.85 and 2.918 Ga (Table 1, analyses A, C) or 3.85 and 2.45 Ga (Table 1, analyses B, D), with each time point being equally likely (uniform prior distribution). The lower limit on the age of crown Cyanobacteria in analyses A and C is based on Mo isotope evidence for oxygen production from the Sinqeni Fm., Pongola Supergroup, South Africa, indicating indirectly the presence of manganese oxidation, a process that would require abundant free oxygen (Planavsky *et al*. 2014). Based on U–Pb zircon ages, rocks containing these Mo signatures were formed at a minimum of 2.940 Ga ± 0.022 Gyr (Hegner *et al*. 1984). Carbon isotope analyses from rocks of similar age, such as Mushandike, Zimbabwe, and Steep Rock, Canada, further support an early presence of oxygen as a result of oxygenic photosynthesis (Nisbet *et al*. 2007; Nisbet and Nisbet 2008). For analyses B and D, the lower limit on the age of crown Cyanobacteria is based on the estimated beginning of the GOE (Bekker *et al*. 2004). The upper limit represents the latest ending of the Late Heavy Bombardment, based on estimates for the end of the strong meteorite bombardment of the moon (Stöffler and Ryder 2001; Chapman *et al*. 2007). Given that the heavy lunar bombardment is estimated to have ended at 3.85 Ga, a similar age may be assumed for Earth given the proximity of the moon. To summarize, calibration 1 followed a uniform distribution from 3.85 to 2.918/2.45 Ga with hard bounds (Table 1).

### Restricting the origin of terminal cell differentiation (calibration 2)

Fossil resting cells, so called akinetes, only present in cyanobacteria capable of heterocyst formation (subsections IV and V; Fig. 1) have been described from *c*. 2.1 Ga Franceville Group, Gabon (Amard and Bertrand‐Sarfati 1997; Sergeev 2009). Based on Sm‐Nd isotopes, two kerogen‐rich shale samples of the Franceville Group were dated 2.099 Ga ± 115 Myr to 2.036 Ga ± 79 Myr, which suggests a conservative minimum constrain for the tree calibration of 1.957 Ga (Bros *et al*. 1992). These fossil akinetes, classified as *Archaeoellipsoides*, have been reported from various intervals through the Proterozoic (Horodyski and Donaldson 1980; Golubic *et al*. 1995; Srivastava 2005); however, the microfossils of the Franceville Group are the earliest of these occurrences. Monophyly of cyanobacteria forming heterocysts and akinetes is confirmed in this study. Combined with the suggestion of a previous study (Tomitani *et al*. 2006), which argued that heterocysts evolved as a response to increasing oxygen levels after the GOE, we assumed that the most recent common ancestor of subsections IV and V cyanobacteria must have originated after the GOE, but prior to 1.957 Ga. We assumed a uniform prior calibration density between 1.957 and 2.45 Ga with 5% prior probability that posterior estimates may lie outside this interval (soft bounds). Although fossil akinetes from the Franceville Group are also known from the Mesoproterozoic (Horodyski and Donaldson 1980; Golubic *et al*. 1995), we wanted to test how strongly calibration 2 affects our age estimates. Therefore, an additional analysis was run without calibration 2. In this case, the following calibrations were applied: (1) calibration 1, uniform prior distribution (min. age: 2.45 Ga and max. age: 3.85 Ga) with hard bounds; (2) calibration 2, missing; and (3) calibration 3, uniform prior distribution (min. age: 2.45 Ga, max. age: 3.33 Ga; soft bounds = 2.5% tail on both sides).

### The evolution of multicellularity – testing two hypotheses (calibration 3)

**Figure 2:**
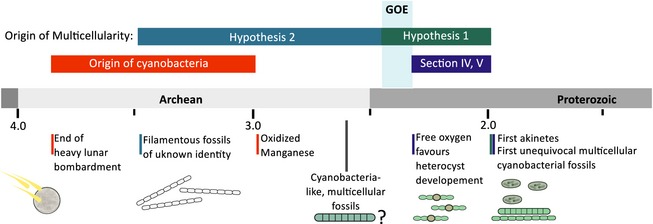
Description of the calibrations used in this study. Age restrictions apply to the origin of Cyanobacteria (calibration 1), the origin of cyanobacterial sections IV and V capable of cellular differentiation (calibration 2) and the origin of multicellularity within cyanobacteria (calibration 3). This study aims to test two hypotheses. Hypothesis 1: Multicellularity originated in cyanobacteria before the Great Oxidation Event (GOE), providing an advantage for cyanobacteria, resulting in higher abundance of those previously scarcely distributed prokaryotes, hence increasing oxygen production. Hypothesis 2: Multicellularity evolved after the GOE, as an adaptation to newly oxidized habitats that became available.

We tested hypotheses on the timing of origin of multicellular cyanobacteria; two hypotheses were specified and tested: whether multicellularity evolved before or after the GOE (Fig. 2). Analyses were conducted restricting the age of the first occurrence of multicellularity at node 69 (result from ancestral character state reconstruction; Schirrmeister *et al*. 2011b), so that this transition occurred with a higher probability either before the GOE (H1) or after the GOE (H2). For H1, 1.957 Ga is set as a minimum age. Rocks of the Franceville Group contain fossil akinetes *Archaeoellipsoides* and ancient relatives of multicellular cyanobacteria referred to as *Archaeorestis* (Amard and Bertrand‐Sarfati 1997). Deposits of similar age from the Duck Creek Dolomite in Western Australia (*c. *2.0 Ga) contain several fossils similar to the multicellular cyanobacterial genus *Lyngbya*, named *Oscillatoriopsis* (Knoll *et al*. 1988). Soft tail probability bounds of 2.5% were implemented. A maximum age of 2.45 Ga was chosen for beginning of the GOE. We assumed a uniform prior distribution with soft bounds (Table 1).

For H2, we set the uniform probability distribution at the estimated beginning of the GOE (2.45 Ga), as estimated by Bekker *et al*. (2004). Filamentous fossils from the 3.42–3.35 Ga found in the Strelley Pool Formation, Australia (Wacey *et al*. 2011; Sugitani *et al*. 2013), Barberton Greenstone Belt, South Africa (Walsh 1992; Westall *et al*. 2001; Westall *et al*. 2006), and possibly earlier (Awramik *et al*. 1988; Schopf and Kudryavtsev 2009) evidence the presence of multicellular prokaryotes. Filament diameters of these fossils are more probabilistic than deterministic as evidence for the presence of cyanobacteria. We chose these occurrences as the basis of a soft maximum bound for the origin of multicellular cyanobacteria. Therefore, prior calibration densities for node 69 are uniformly distributed between 3.33 and 2.45 Ga, and minimum and maximum ages refer to the 95% probability bounds (Table 1).

## Results

With the aim of inferring the evolutionary history of cyanobacteria in respect of their fossil record, we concatenated 756 highly conserved protein sequences (Schirrmeister *et al*. 2015, appendices S1, S2) that were previously identified within 65 cyanobacterial taxa based on similarity searches. Cyanobacterial phylogenies based on maximum likelihood methods are presented in Figure 1 (see also Fig. S1; Schirrmeister *et al*. 2015, appendix S3).

### Comparison of phylogenetic reconstructions based on genome and ribosomal data

For direct comparison of our phylogenomic tree (Fig. 1) to trees based on ribosomal genes, we reconstructed a maximum likelihood phylogeny based on concatenated 16S and 23S rRNA sequences of the taxa used in this study (Fig. S1). While 100% maximum likelihood bootstrap (BS) values supported just 24 of 64 branches of the ribosomal tree, 100% bootstrap support was inferred for 57 of 64 branches of the genomic tree. Several forms of genera appear polyphyletic, such as *Synechococcus*,* Geitlerinema* and *Pseudanabaena*. Across the phylogenomic tree, six major clades could be identified with full support from bootstrap resamplings. Assuming that *Gloeobacter* groups closest to a eubacterial outgroup, as previously suggested based on ribosomal (Schirrmeister *et al*. 2011b), and phylogenomic data (Blank and Sanchez‐Baracaldo 2010; Shih *et al*. 2013), *Synechococcus* isolated from hot springs are the first group to split from the rest of Cyanobacteria, followed by an early branching ‘Clade 1’ of unicellular and multicellular cyanobacteria comprising *Synechocystis* sp. PCC 9635, and *Pseudanabeana* sp. PCC 7367, PCC 7704 and PCC 7904 (Clade C, Figs S1, S2). Unicellular *Acaryochloris marina* MBIC11017, *Acaryochloris* sp. CCMEE 5410 and *Thermosynechococcus elongatus* BP‐1 form a separate ‘Clade 2’. ‘Clade 3’ comprises early branching unicellular and multicellular taxa, including marine picocyanobacteria and filamentous *Prochlorothrix hollandica* PCC 9006. ‘Clade 4’ includes a group of multicellular taxa, such as *Arthrospira maxima CS‐328* and *A. platensis* NIES‐39, *Oscillatoria nigro‐viridis* PCC 7112*, Planktothrix agardhii* NIVA‐CYA 34 and *Trichodesmium erythraeum* IMS101. ‘Clade 5’ and ‘Clade 6’ form sister clades. ‘Clade 5’ contains subsections IV and V cyano‐bacteria, unicellular *Chroococcidiopsis* sp. PCC 8201 and *Chroococcidiopsis thermalis* PCC 7203, and multicellular *Limnothrix redekei* PCC 9416. Unicellular *Chamaesiphon minutus* PCC 6605 and multicellular *Crinalium epipsammun* PCC 9333, which are included in this clade in the ribosomal tree, are grouped separately with good bootstrap support (BS: 75%) in the phylogenomic tree. Phylogenomic data fully support monophyly of subsections IV and V cyanobacteria capable of cellular differentiation. ‘Clade 6’ comprises the majority of the subsections I to III cyanobacteria (except marine picocyanobacteria, hot spring *Synechococcus* and early‐branching multicellular taxa) and is well supported in the phylogenomic tree, but not resolved in the ribosomal tree (BS: 41%).

Clades reconstructed in this study were compared to previous studies (Table 2; Schirrmeister *et al*. 2011b; Shih *et al*. 2013; Sanchez‐Baracaldo *et al*. 2014). Clades 1–6 (this study) can be directly compared to clades (A–C, E, F) reconstructed by Shih *et al*. (2013). Early branching multicellular taxa described here as Clade 1 can be found as Clade C in Schirrmeister *et al*. (2011b), but were missing in other studies (Blank and Sanchez‐Baracaldo 2010; Sanchez‐Baracaldo *et al*. 2014). Clades 2 and 3 (this study) comprising mostly unicellular taxa, including picocyanobacteria and clades 4–6 (this study), are comparable to Clade AC and Clade E in Schirrmeister *et al*. (2011b). In Sanchez‐Baracaldo *et al*. (2014), our Clade 3 is partly recovered as Marine SynPro, clades 4 and 5 were recovered as clade PNT, while Clade 6 is comparable to their SPM.

**Table 2:**
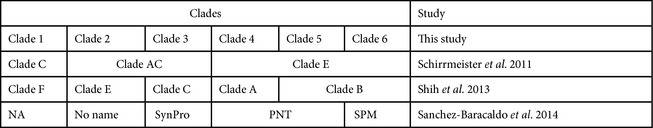
Clade comparison to previous studies

### Ancestral character state reconstruction

**Figure 3:**
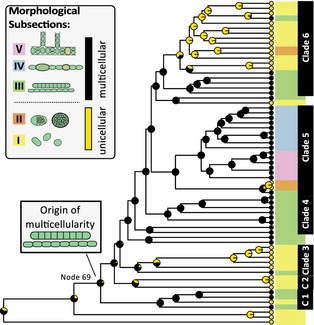
Ancestral character state reconstruction to infer the evolution of multicellularity. Ancestral character states inferred from maximum likelihood analyses assuming asymmetrical transition rates between states were plotted on an ultrametric maximum likelihood tree of cyanobacteria. Pie charts on nodes display reconstructed ancestral character states, where black depicts multicellular and yellow unicellular growth states. Modern cyanobacterial taxa are displayed in coloured boxes, which indicate their taxonomic classification according to Rippka *et al*. (1979). Taxa belonging to unicellular subsections I and II are displayed with a yellow and orange background, respectively, whereas multicellular cyanobacteria from subsections III, IV and V are shown in green, blue and pink boxes, respectively. C1 and C2 refer to clades 1 and 2. Multicellularity evolved early during cyanobacterial history was lost several times and regained in two lineages.

The evolution of multicellularity was reconstructed using a maximum likelihood and Bayesian inference. An evolutionary model assuming equal transition rates resulted in rates of 0.7575 (standard deviation = 0.1978). For character state reconstructions using an asymmetrical rate model, the estimated rates were q01 = 0.5768 (standard error = 0.3147) and q10 = 0.8254 (standard error = 0.2205), where q01 defines the transition to multicellularity and q01 the loss of multicellularity. Additionally, different uniform prior distributions for the estimation of transition rates were tested during the Bayesian analyses of ancestral character states. Changing the widths of the uniform distribution in several runs supported an early transition to multicellularity even further (Fig. S3, online Supporting Information). All MCMC runs exhibited evidence of sufficient mixing. For all analyses, accepted changes to the chains had values of approximately 30% at the time of convergence. Results of all analyses suggest a very early transition to multicellularity at ‘node/branch 69’ (Fig. 3; Fig. S3; Table S3). Across the phylogeny, multicellularity is subsequently lost at least five times and regained on one, possibly two, branch leading to *Leptolyngbya* sp. PCC 7376 and *Spirulina subsalsa* PCC 9445. Hence, the majority of modern cyanobacteria are descendants of an ancient multicellular lineage.

### Divergence time estimation

Divergence time analyses of the phylogenomic data set employed three calibration points that have been refined from previously suggested calibrations for dating analyses of Cyanobacteria (Schirrmeister *et al*. 2013) and comprised (C1) the origin of Cyanobacteria, (C2) the origin of cellular differentiation as found in subsections IV and V based on fossilized 2.1 Ga akinetes and (C3) the origin of multicellularity at node 69 (Fig. 3). Compared to previous analyses, which could not resolve the phylogenetic placement of the most recent common ancestor (MRCA) of subsections IV and V, our phylogenomic data allow us to recover monophyly of this group, hence enabling a more precise calibration of this divergence event (C2). For the origin of multicellularity (C3), two scenarios were tested, including: (1) an origin of multicellularity after the GOE as an adaptation to newly formed (oxygenated) habitats (Hypothesis 1); or (2) an origin of multicellularity before the GOE, where advantages of multicellularity could have resulted in an increasing abundance of cyanobacteria, hence increasing net oxygen production and causing the GOE (Hypothesis 2; Fig. 2).

**Figure 4:**
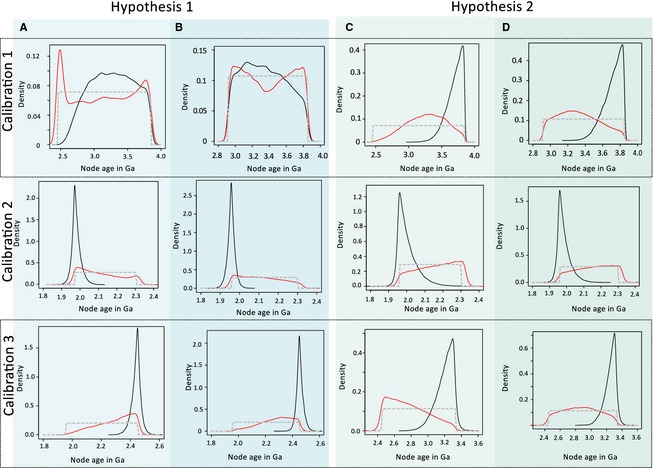
Prior and posterior age estimates for nodes that have been calibrated. User‐specified (grey) and effective prior age densities (red), as well as posterior age estimates (black) for the origin of cyanobacteria (calibration 1), the origin of sections IV and V cyanobacteria (calibration 2) and the origin of multicellularity (calibration 3). Compared are two hypotheses, each with (A, C) a wider (3.85–2.45 Ga) and (B, D) a narrower root calibration (3.85–2.958 Ga). The posterior age estimates for the origin of multicellularity (calibration 3) are in all cases shifted towards the older bound of the effective priors.

Divergence time results are strongly affected by parameter choice if non‐uniform prior calibration densities, such as lognormal, are employed (Warnock *et al*. 2012, 2015); therefore, only uniform distributions were applied in this study. Additionally, Warnock *et al*. (2012, 2015) pointed out that programs for molecular clock analyses will truncate prior calibrations so that prior age distributions agree with the occurrences of divergence events in the phylogeny. Therefore, the joint (effective) calibration priors may differ substantially from the user‐specified priors. Our analyses run without molecular data showed that our originally uniform priors (Fig. 4; dashed curve) resulted in non‐uniform effective priors (Fig. 4; red curve). Age ranges of specified and effective priors were similar, however, and could be accepted for the divergence time analyses. For the cyanobacterial stem lineage (calibration 1; Fig. 4), posterior age estimates were almost uniformly distributed in Hypothesis 1 and shifted towards an origin of cyanobacteria prior 3.5 Ga for Hypothesis 2. In all analyses, subsections IV and V capable of cellular differentiation (calibration 2; Fig. 4) have a higher probability of origination close to the minimum age range of our specified prior calibration (1.9–2.1 Ga). However, if this node was not used for calibration, a younger age is estimated (0.8–1.6 Ga; Fig. S4). In this case, similar posterior ages are estimated for node 3 and the root, but with broader 95% probability distributions. In all analyses, the posterior probabilities of the origin of multicellularity (calibration 3; Fig. 4) support an age close to the maximum of the prior age calibration.

**Figure 5:**
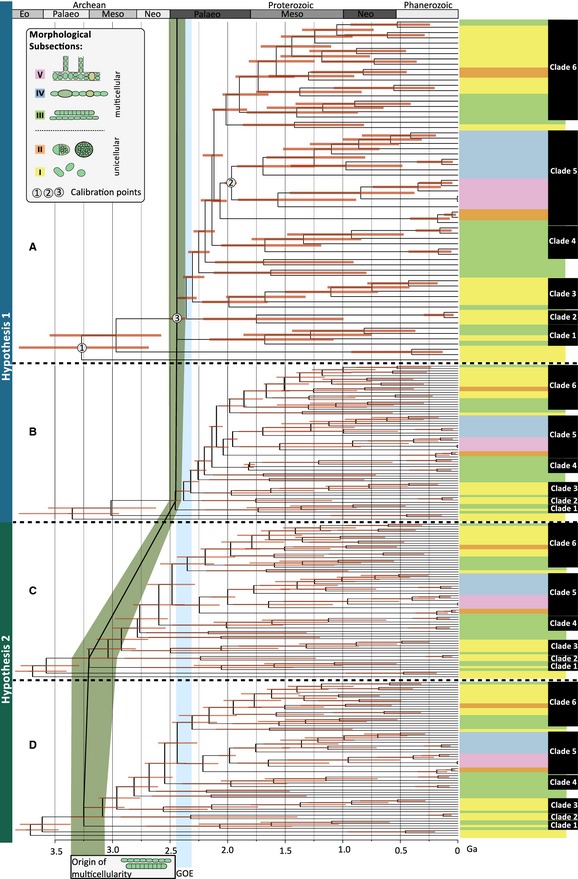
Divergence time of cyanobacteria. Divergence times were reconstructed using a relaxed molecular clock. Two hypothesis have been tested, where multicellularity is assumed to have originated after the Great Oxidation Event (GOE; Hypothesis 1; A, B) or before the GOE (Hypothesis 2; C, D). The origin of cyanobacteria (root) was calibrated between the end of the late heavy bombardment and (A, C) the onset of the GOE, or (B, D) the first traces of oxygen at 2.958 Ga. In all four analyses, the cyanobacterial multicellularity is estimated to originate before the GOE. Nodes for calibrations 1–3 are marked in the first phylogenomic tree. Trees are ultrametric versions of the maximum likelihood tree presented in Figure 1. Colours refer to different morphological subsections of cyanobacteria. From top to bottom, the following taxa are displayed in each tree: (Clade 6) *Leptolyngbya* sp. PCC 7376, *Synechococcus* sp. PCC 7002, *Cyanobacterium aponinum* PCC 10605, *Cyanobacterium stanieri* PCC 7202, *Synechocystis* sp. PCC 6803 substrain PCC‐N, *Cyanothece* sp. PCC 7424, *Microcystis aeruginosa* NIES‐843, *Cyanothece* sp. PCC 8801, *Crocosphaera watsonii* WH 8501, *Xenococcus* sp. PCC 7305, *Stanieria cyanosphaera* PCC 7437, *Gloeocapsa* sp. PCC 73106, *Halothece* sp. PCC 7418, *Dactylococcopsis salina* PCC 8305, *Spirulina subsalsa* PCC 9445, *Moorea producens* 3L,*Symploca* sp. PCC 8002, *Coleofasciculus chthonoplastes* PCC 7420, *Microcoleus* sp. PCC 7113; *Crinalium epipsammum* PCC 9333, *Chamaesiphon minutus* PCC 6605; (Clade 5) ‘*Nostoc azollae*’ 0708, *Cylindrospermopsis raciborskii* CS‐505, *Anabaena cylindrica* PCC 7122, *Cylindrospermum stagnale* PCC 7417, *Microchaete* sp. PCC 7126, *Anabaena variabilis* ATTC 29413, *Nostoc* sp. PCC 7120, *Nodularia spumigena* CCY9414, *Calothrix* sp. PCC 6303, *Fischerella muscicola* PCC 7414, *Fischerella thermalis* PCC 7521, *Chlorogloeopsis fritschii* PCC 9212, *Chlorogloeopsis fritschii* PCC 6912, *Mastigocladopsis repens* PCC 10914, *Chroococcidiopsi*s sp. PCC 8201, *Chroococcidiopsis thermalis* PCC 7203, *Limnothrix redekei* PCC 9416; (Clade 4) *Arthrospira platensis* NIES‐39, *Arthrospira maxima* CS‐328, *Planktothrix agardhii* NIVA‐CYA 34, *Trichodesmium erythraeum* IMS101, *Microcoleus vaginatus* FGP‐2, *Oscillatoria nigro‐viridis* PCC 7112; *Geitlerinema* sp. PCC 7407, *Leptolyngbya* sp. PCC 73110, *Geitlerinema* sp. PCC 8501, *Nodosilinea nodulosa* PCC 7104; (Clade 3) *Synechococcus* sp. WH 8102, *Synechococcus* sp. CC9605, *Synechococcus* sp. CC9311, *Cyanobium gracile* PCC 6307, *Synechococcus elongatus* PCC 6301, *Prochlorothrix hollandica* PCC 9006; (Clade 2) *Acaryochloris marina* MBIC11017, *Acaryochloris* sp. CCMEE 5410, *Thermosynechococcus elongatus* BP‐1; and (Clade 1) *Pseudanabaena* sp. PCC 7904, *Pseudanabaena* sp. PCC 7704, *Synechocystis* sp. PCC 9635, *Pseudanabaena* sp. PCC 7367; *Synechococcus* sp. JA‐3‐3Ab, *Synechococcus* sp. JA‐2‐3B'a(2‐13), *Gloeobacter violaceus* PCC 7421.

Figure 5 presents the results of the divergence time analyses comparing all four analyses (Table 1). Posterior node age estimates show large 95% confidence intervals, which prevents a precise dating of divergence events. Nevertheless, all analyses, irrespective of the prior assumptions applied for ‘node 69’, point to an origin of multicellularity in cyanobacteria before the GOE (Fig. 5). The majority of modern cyanobacterial clades (clades 2–6) have evolved after the GOE. Furthermore, our results agree with the previous estimates of an origin of marine picocyanobacteria around the end of the Mesoproterozoic (Sanchez‐Baracaldo *et al*. 2014), although confidence intervals are too broad to specify dates, further. In comparison with the previous analyses based on 16S rRNA data (Schirrmeister *et al*. 2013), confidence intervals are smaller in general, particularly those of deep node ages.

Different widths for the calibration of the root (A, C vs. B, D in Fig. 5) did not affect age estimates for node 69, whereas in Hypothesis 1 a calibration of the root between 3.85 and 2.958 resulted in a mean estimate of 235 million years older age for node 69 with wider posterior probability distribution.

## Discussion

### Improved data set to reconstruct the evolutionary history of Cyanobacteria

Applying 16S rRNA sequence data and a set of different calibration assumptions, a previous study by Schirrmeister *et al*. (2013) estimated divergence times that pointed towards an origin of cyanobacteria in the Archean Eon, with multicellularity more likely originating before the GOE. However, these results were based on a minimal data set of 16S ribosomal RNA sequences (1077–1090 nucleotide sites). Although the occurrence of both variable sites and highly conserved sites has made 16S rRNA sequences an ideal tool to recover phylogenetic relationships on a phylum level (Woese 1987), it comprises merely 1400 nucleotide sites with only half of them susceptible to change in phylum Cyanobacteria. Therefore, the chances are high that phylogenetic information may have been lost due to multiple substitutions at the same nucleotide site (saturation). Although evolutionary models try to account for multiple hits, over time it will become more likely that sequences saturate, which is a problem particularly for nucleotide sequences and deep branches (Xia *et al*. 2003).

Another study by Shih and Matzke (2013) recovered a much more recent origin of living cyanobacterial taxa concluding that an ancient cyanobacterial stem lineage must be responsible for late Archean oxygen levels. Although their cross‐calibration, cross‐bracing technique for duplication events is innovative, their results are based on merely two subunits of one enzyme (*c*. 986 amino acids). The ATPase subunits resulted from an ancient duplication event, which occurred before the last common ancestor of all living taxa and has therefore been used in the past to reconstruct the Tree of Life (Iwabe *et al*. 1989). Aside from potential sequence saturation that may have occurred in these sequences, the reconstructed phylogenies depict the evolution of an enzyme and may result in strongly misleading inferences for species evolution. Additionally, calibrations of tip clades have been claimed to be unsuitable for the accurate estimation of deep divergence events (Duchene *et al*. 2014).

In this study, the use of an impressively large data set (197 766 amino acid sites) helped to overcome the limitations previous analyses might have had, not only substantially improving the statistical support across cyanobacterial phylogeny in comparison with single‐gene analyses, but also corroborating the hypotheses of an early origin of multicellularity in Cyanobacteria (Schirrmeister *et al*. 2011b, 2013).

### Comparison of cyanobacterial cell sizes to Precambrian microfossils

Modern cyanobacteria exhibit a wide range of cell sizes which, on average, exceeds sizes found among most prokaryotes (<2 μm; Castenholz 2001). Microfossils from Proterozoic and Archean deposits comprise multicellular forms, such as septate and non‐septate filaments, and unicellular remains, such as spheres and rods. Often, sizes of unicellular taxa exceed those of fossilized filaments, as observed in deposits, such as Dismal Lake, Gunflint Chert, Belcher Supergroup and Barberton Supergroup (Hofmann 1976; Horodyski and Donaldson 1980; Lanier 1989; Walsh 1992). In early Archean deposits, such as in South Africa (3.33–3.46 Ga), fossil filament sizes do not exceed 5 μm (Walsh 1992; Westall *et al*. 2001). While an occurrence of larger tubes (10–20 μm) has been observed in 3.4 Ga deposits from Western Australia, these show few characteristics to link them to modern prokaryotic taxa. The first larger, well‐preserved, multicellular morphotypes with cell widths of up to 28 μm can be observed in the 2.6 Ga Transvaal Group (Altermann and Schopf 1995). Filaments of this deposit have been compared to cyanobacterial morphotypes. In *c. *2.0 Ma deposits from Western Australia, filaments of large size (63 μm in width) have been found and classified as *Oscillatoriopsis majuscule* (Knoll *et al*. 1988). Fossils of such sizes resemble modern filamentous cyanobacterial sizes observed in the form of genera *Lyngbya*,* Oscillatoria* and *Trichodesmium* (Castenholz 2001).

Our results show that multicellularity evolved very early among cyanobacteria and divergence time estimations (Fig. 5) confirm previous suggestions of an origin of multicellularity prior to the GOE, independently of whether the 2.0 Ga fossil akinete calibration is used. The assumptions that: (1) simple filamentous cyanobacterial taxa were present in 2.0‐Gyr‐old deposits; and (2) cyanobacteria were responsible for the accumulation of oxygen during the Archean, indicates an existence of multicellular cyanobacteria before the GOE. This has some significance for the interpretation of filamentous fossils as cyanobacteria based on their morphology, including those described from a range of sites at 2.0 Ga (Knoll *et al*. 1988; Lanier 1989; Amard and Bertrand‐Sarfati 1997) and potentially from the 2.6 Ga Transvaal Supergroup (Altermann and Schopf 1995) or even older (Awramik *et al*. 1988; Schopf and Kudryavtsev 2009).

Geochemical evidence, based on Mo and Fe isotopes, supports an early evolution of oxygenic photosynthesis (Nisbet and Fowler 2011). More than 2.83‐Gyr‐old reef systems from Mushandike, Zimbabwe, and Steep Rock, Canada, seem to have been formed in oxic waters and support the hypotheses of Archean oxygen oases that were based on the absence of mass‐independent sulphur fractionation (Ohmoto *et al*. 2006). Isotopes from organic carbon in these rocks, additionally, point towards cyanobacterial RuBisCO I activity in those deposits and support an origin of cyanobacteria in the Archean (Nisbet *et al*. 2007; Nisbet and Fowler 2011).

### Benefits of cyanobacterial multicellularity and its potential consequences for the GOE

The origin of Cyanobacteria has been widely discussed in the past and yet is still far from being resolved. Previous hypotheses have argued for an origin of Cyanobacteria shortly before the GOE (Kirschvink and Kopp 2008), followed by a rapid accumulation of oxygen as observed during the GOE. Alternatively, several studies have found evidence of free oxygen and, thus, inferred the presence of cyanobacteria, hundreds of millions of years prior to the GOE, possibly as early as 3.0 Ga (Nisbet *et al*. 2007; Nisbet and Nisbet 2008; Crowe *et al*. 2013; Lyons *et al*. 2014). The delay in the accumulation of free oxygen until the GOE has been explained by a strongly reducing atmosphere where initially produced oxygen was captured by oxygen sinks, such as reduced volcanic gases (Catling and Claire 2005). It has been shown that Fe (II) inhibits growth in *Synechococcus* cultures (Swanner *et al*. 2015). In Archean environments, Fe (II) toxicity could have prohibited oxygenic photosynthesis and subsequently allowed for only little oxygen production. Although reducing gases would have influenced oxygen levels, studies fail to take into account the importance of cyanobacterial diversity and population sizes. As the only biological source of oxygen during the early Precambrian, cyanobacterial fitness and ecological success should have had a profound impact on the amount of free net oxygen in Earth's ocean and atmosphere. We suggest that the origin of multicellularity was a major transition in the history of cyanobacteria, forming new units to be subject to selection, and offering the potential to increase cyanobacterial abundance, consequently initiating the GOE. Furthermore, multicellularity could have provided novel possibilities for cyanobacterial diversification and adaptation to new oxygenated habitats.

**Figure 6:**
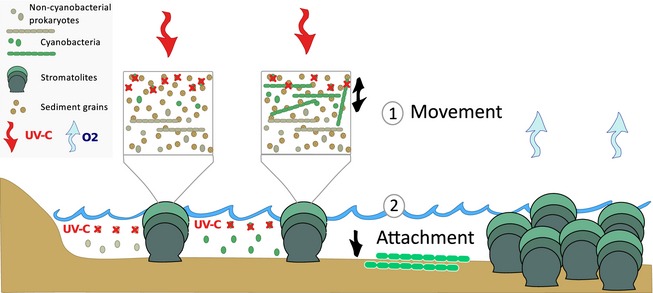
Illustration of cyanobacterial evolution leading towards the GOE. UV‐C radiation (below 290 nm) might have proved a major challenge for amotile unicellular cyanobacteria. The development of multicellularity will have provided two major advantages to a mat community: (1) the ability to move within the bacterial mat according to light requirements and/or lethal UV‐C avoidance; and (2) better attachment during the initial phase of mat development. These advantages of multicellularity in combination with the energetically higher efficiency of oxygenic photosynthesis may have led to a greater abundance of cyanobacterial‐dominated stromatolites and indirectly resulted in higher O_2_ production towards the end of the Archean.

The origin of multicellularity may have had manifold advantages in an Archean environment. As we have already observed, metabolic rates seem to increase superlinearly with size in heterotrophic bacteria (DeLong *et al*. 2010). Similarly, increased sizes of multicellular cyanobacteria may have resulted in higher metabolic rates, hence, higher photosynthetic activity and increased net oxygen production. Additionally, multicellular growth may have provided competitive advantages that resulted in a higher growth success (Fig. 6). During cyanobacterial evolution, UV radiation must have been a strong selective pressure resulting in several cyanobacterial taxa, today, that have developed a capability to protect themselves from UV‐A and UV‐B using pigments, such as scytonemin and shinorine (Sinha and Häder 2008; Balskus and Walsh 2010; Calteau *et al*. 2014; Rastogi *et al*. 2014). Although it has been suggested that sulphur may have provided UV shielding during the Archean (Ueno *et al*. 2009), UV radiation could have been problematic for photosynthetic organisms, as they rely on sunlight as energy source but, at the same time, need to avoid lethal radiation. Prior to the GOE, before an ozone shield was established, additional ultraviolet (UV) radiation between 200 and 300 nm (UV‐C) would have had lethal effects on organisms (Kasting and Catling 2003). The ability to move within the microbial mat, a characteristic that has been described for multicellular cyanobacteria, could have been of great advantage to avoid lethal radiation. In modern mat communities, multicellular cyanobacteria, such as *Oscillatoria* and *Phormidium*, move vertically to position themselves in optimal light (Stal 1995). Furthermore, experiments have demonstrated the movement of filamentous cyanobacteria to avoid UV light, where prevention of movement resulted in UV‐induced inhibition of photosynthesis (Kruschel and Castenholz 1998). Furthermore, multicellularity may have been advantageous during initial substrate attachment at the beginning of mat formation (Fig. 6). Larger surface area of filamentous bacteria increases contact to the substrate which could have resulted in improved attachment (Young 2006). Filamentous cyanobacteria of the genus *Oscillatoria* have been associated with initial substrate colonization during mat formation (Stal *et al*. 1985). Improved attachment of filamentous cyanobacteria at the end of the Archean could have increased mat formation and consequently success of cyanobacterial taxa associated with those microbial communities. Additionally, multicellularity in cyanobacteria enabled further evolutionary innovations. Cellular differentiation in Cyanobacteria, for example, can only evolve in a multicellular setting (Rossetti *et al*. 2010). Therefore, potential advantages of multicellularity such as: (1) movement and (2) improved substrate attachment in combination with potential increases in metabolic rates could have been the trigger for evolutionary success of cyanobacteria at the end of the Archean initiating the oxygenation of Earth.

## Conclusion

Cyanobacteria originated in the mid‐Archean, long before the GOE, with multicellular taxa evolving towards the end of the Archean. An observed delay in oxygen accumulation until the GOE could have been associated not only with oxygen sinks that remove oxygen, but possibly also with decreased evolutionary success of early Cyanobacteria. Multicellularity provides selective advantage during early Earth history including: (1) movement within microbial mats to avoid lethal UV‐C dosages; and (2) improved attachment to surfaces during initial mat formation that may have substantially increased the success of cyanobacterial‐dominated mat communities and enabled adaptation to novel habitats. Comparisons of modern cyanobacterial taxa to Precambrian microfossils support the inferred presence of multicellular cyanobacteria before the GOE, but the fossil record does not provide evidence for multicellular taxa among the earliest unequivocal fossil assemblages at 3.33–3.45 Ga.

## Supporting information


**Fig. S1.** Phylogenetic Maximum Likelihood tree based on ribosomal genes.

**Fig. S2.** Maximum Likelihood tree displaying node numbers.

**Fig. S3.** Character state reconstruction at node 69 using MCMC runs.

**Fig. S4.** Divergence time reconstructions excluding calibration 2.

**Table S1.** Cyanobacterial taxa used in this study.

**Table S2.** Prior transition rates for the character state reconstruction applying Bayesian inference.

**Table S3.** Reconstructed ancestral character states.
