# Supplementary figures and images for: Cyanobacteria and the Great Oxidation Event: evidence from genes and fossils
Source: Palaeontology. 2015 Jun 23;58(5):769–85. doi: 10.1111/pala.12178 (PMC4755140; doi:10.1111/pala.12178)

**Morphological Subsections:**

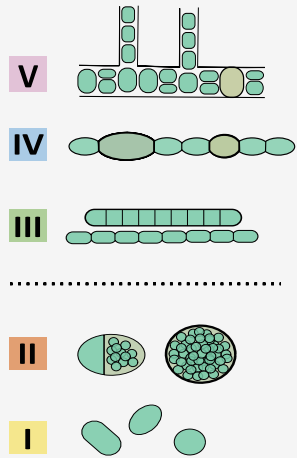

# multicellular

unicellular

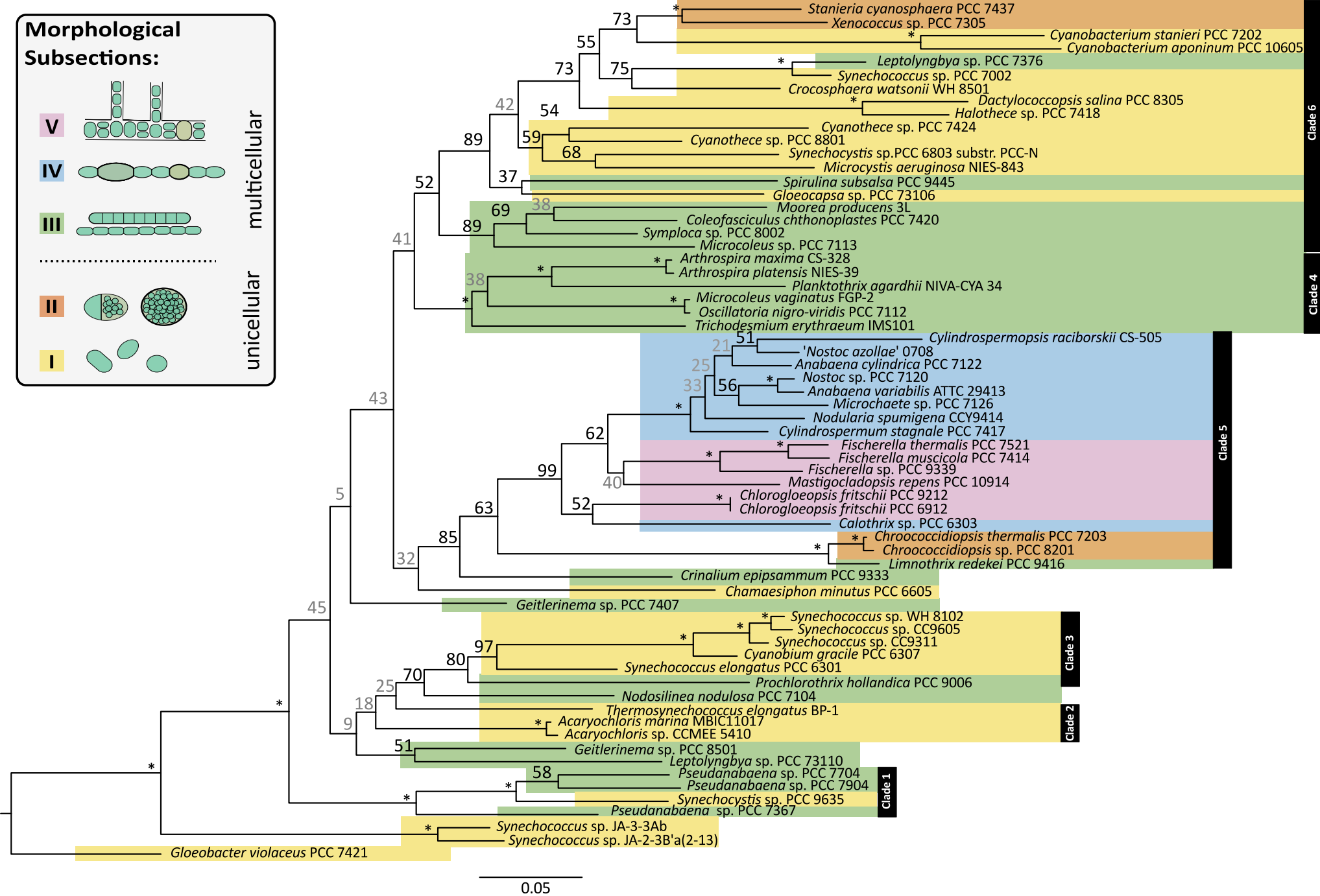

Supplement: Supplementary file 1 — Fig. S1. Phylogenetic Maximum Likelihood tree based on ribosomal genes. [file PALA-58-769-s001.pdf]

# Morphological

## Subsections:

V

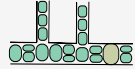

IV

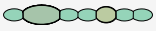

III

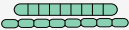

II

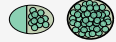

I

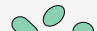

multicellular

unicellular

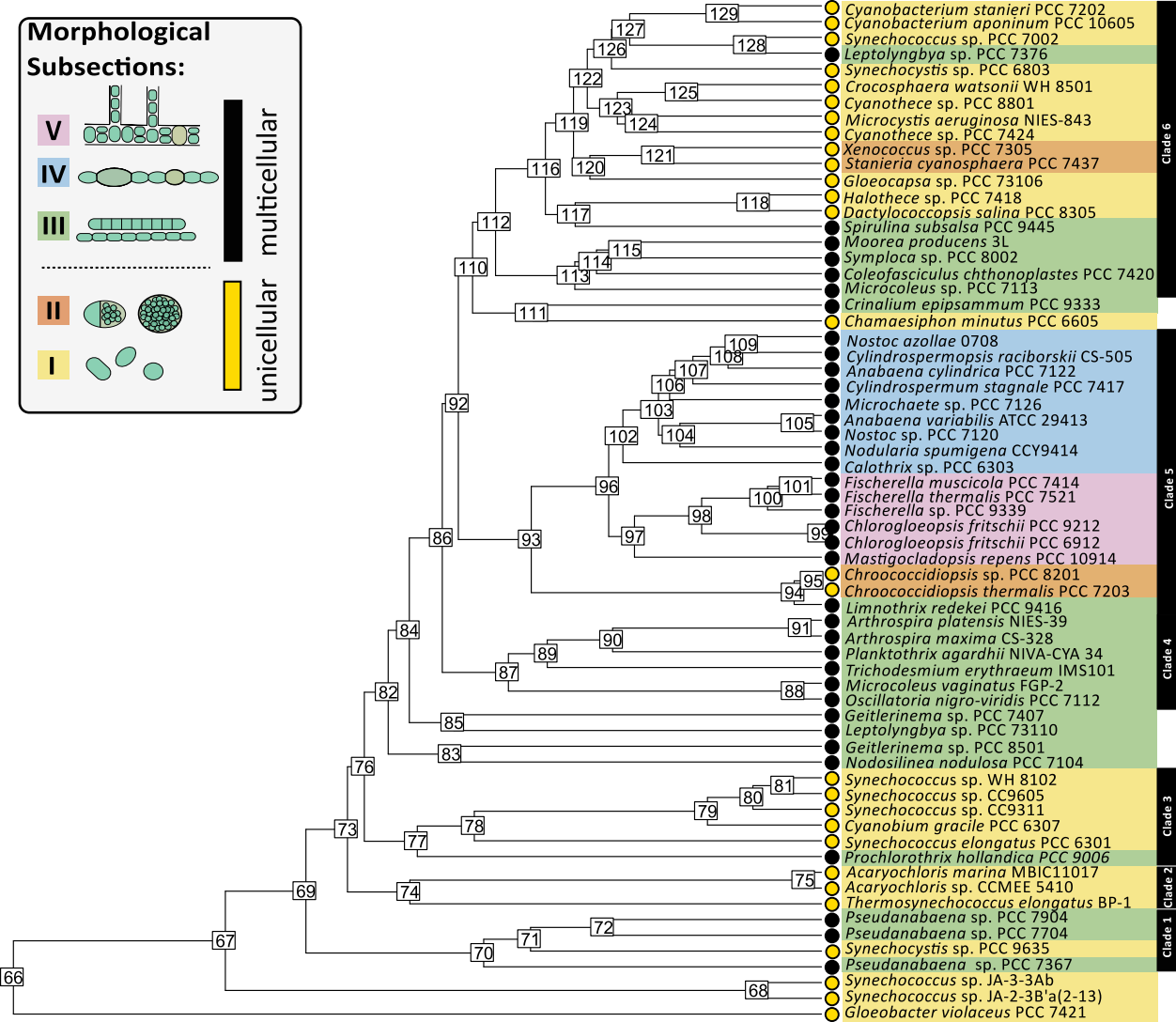

Supplement: Supplementary file 2 — Fig. S2. Maximum Likelihood tree displaying node numbers. [file PALA-58-769-s002.pdf]

A)

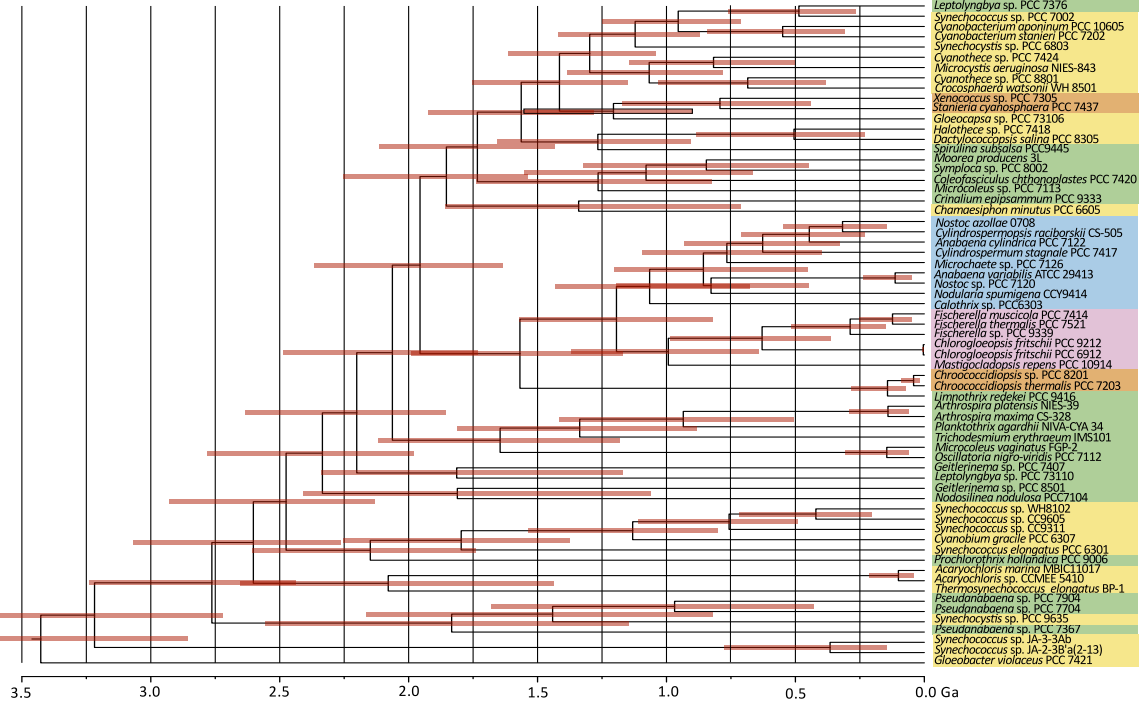

B)

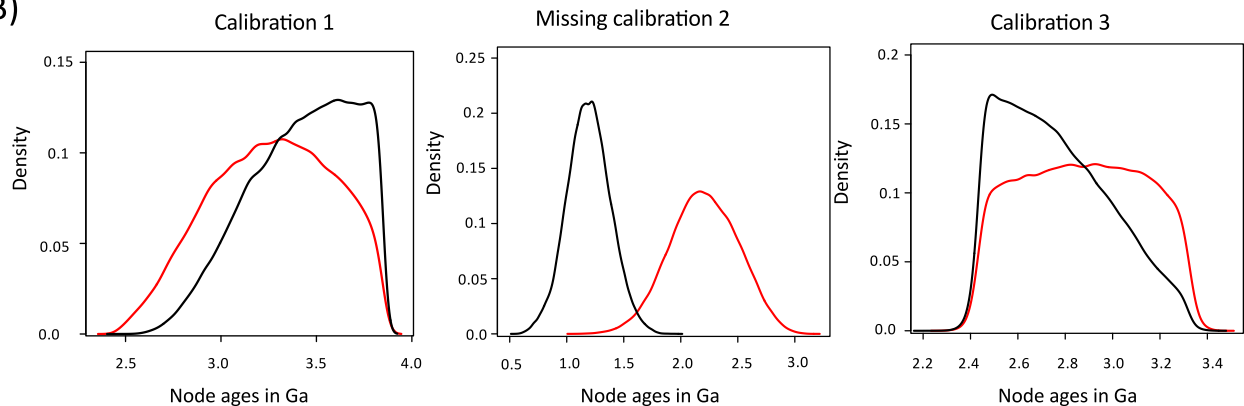

Supplement: Supplementary file 4 — Fig. S4. Divergence time reconstructions excluding calibration 2. [file PALA-58-769-s004.pdf]
