# Supplementary material for: Cyanobacteria and the Great Oxidation Event: evidence from genes and fossils
Source: Palaeontology. 2015 Jun 23;58(5):769–85. doi: 10.1111/pala.12178 (PMC4755140; doi:10.1111/pala.12178)
Supplement: Supplementary file 3 — Fig. S3. Character state reconstruction at node 69 using MCMC runs. [file PALA-58-769-s003.pdf]

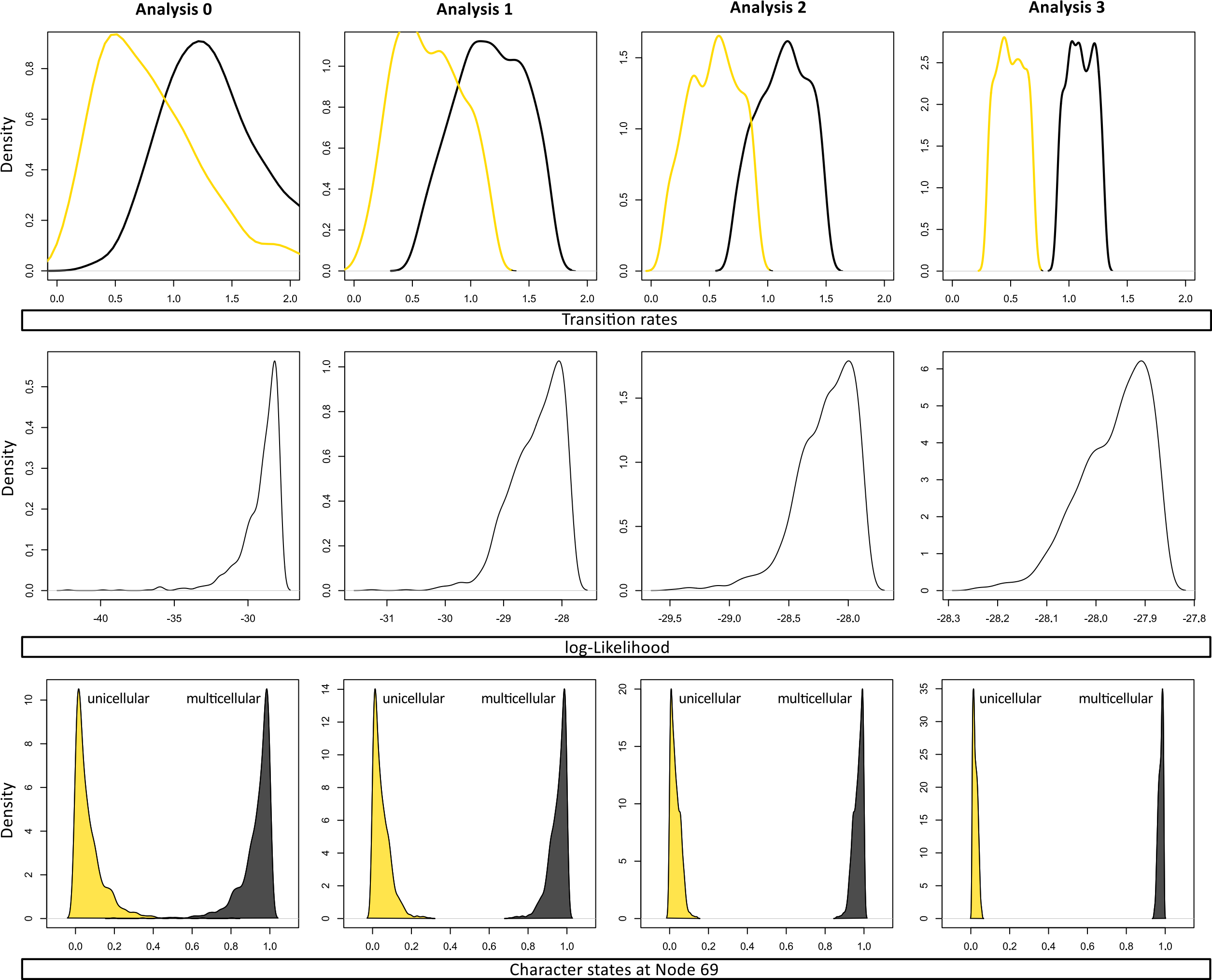

Analysis 0
Analysis 1
Analysis 2
Analysis 3
Density
Transition rates
log-Likelihood
unicellular
multicellular
Character states at Node 69
