# Supplementary material for: Cyanobacteria and the Great Oxidation Event: evidence from genes and fossils
Source: Palaeontology. 2015 Jun 23;58(5):769–85. doi: 10.1111/pala.12178 (PMC4755140; doi:10.1111/pala.12178)
Supplement: Supplementary file 5 — Table S1. Cyanobacterial taxa used in this study. [file PALA-58-769-s005.docx]

| **Taxon name** | **Taxon ID** | **Accession** | **state** | **Taxon name** | **Taxon ID** | **Accession** | **state** |
| --- | --- | --- | --- | --- | --- | --- | --- |
| **Subsection I** |  |  |  | **Subsection III** |  |  |  |
| *Acaryochloris marina* MBIC11017 | 329726 | CP000828 | 0 | *Geitlerinema* sp. PCC 8501 | this study |  | 1 |
| *Acaryochloris* sp. CCMEE 5410 | 310037 | AFEJ01000000 | 0 | *Leptolyngbya* sp. PCC 7376 | 111781 | CP003946 | 1 |
| *Chamaesiphon minutus* PCC 6605 | 1173020 | CP003600 | 0 | *Leptolyngbya* sp. PCC 73110 | this study |  | 1 |
| *Crocosphaera watsonii* WH 8501 | 165597 | AADV02000000 | 0 | *Limnothrix redekei* PCC 9416 | this study |  | 1 |
| *Cyanobacterium stanieri* PCC 7202 | 292563 | CP003940 | 0 | *Microcoleus* sp. PCC 7113 | 1173027 | CP003630 | 1 |
| *Cyanobium gracile* PCC 6307 | 292564 | CP003495 | 0 | *Microcoleus vaginatus* FGP-2 | 756067 | AFJC01000000 | 1 |
| *Cyanobacterium aponinum* PCC 10605 | 755178 | CP003947 | 0 | *Moorea producens* 3L | 489825 | AEPQ01000000 | 1 |
| *Cyanothece* sp. PCC 7424 | 65393 | CP001291 | 0 | *Nodosilinea nodulosa* PCC 7104 | 118166 | ALVP01000000 | 1 |
| *Cyanothece* sp. PCC 8801 | 41431 | CP001287 | 0 | *Oscillatoria nigro-viridis* PCC 7112 | 179408 | CP003614 | 1 |
| *Dactylococcopsis salina* PCC 8305 | 13035 | CP003944 | 0 | *Planktothrix agardhii* NIVA-CYA 34 | 213631 | AVFT00000000 | 1 |
| *Gloeobacter violaceus* PCC 7421 | 251221 | BA000045 | 0 | *Prochlorothrix hollandica* PCC 9006 | 317619 | ANKN00000000 | 1 |
| *Gloeocapsa* sp. PCC 73106 | 102232 | ALVY01000000 | 0 | *Pseudanabaena* sp. PCC 7367 | 82654 | CP003592 | 1 |
| *Halothece* sp. PCC 7418 | 65093 | CP003945 | 0 | *Pseudanabaena* sp. PCC 7704 | this study |  | 1 |
| *Microcystis aeruginosa* NIES-843 | 449447 | AP009552 | 0 | *Pseudanabaena* sp. PCC 7904 | this study |  | 1 |
| *Synechocystis* sp. PCC 6803 substrain PCC-N | 1080229 | AP012277 | 0 | *Spirulina subsalsa* PCC 9445 | 1173029 | ALVR01000000 | 1 |
| *Synechocystis* sp. PCC 9635 | this study |  | 0 | *Symploca* sp. PCC 8002 | this study |  | 1 |
| *Synechococcus elongatus* PCC 6301 | 269084 | AP008231 | 0 | *Trichodesmium erythraeum* IMS101 | 203124 | CP000393 | 1 |
| *Synechococcus* sp. PCC 7002 | 32049 | CP000951 | 0 | **Subsection IV** |  |  |  |
| *Synechococcus* sp. CC9311 | 64471 | CP000435 | 0 | *Anabaena cylindrica* PCC 7122 | 272123 | CP003659 | 1 |
| *Synechococcus* sp. CC9605 | 110662 | CP000110 | 0 | *Anabaena variabilis* ATTC 29413 | 240292 | CP000117 | 1 |
| *Synechococcus* sp. JA-2-3B'a(2-13) | 321332 | CP000240 | 0 | *Calothrix* sp. PCC 6303 | 1170562 | CP003610 | 1 |
| *Synechococcus* sp. JA-3-3Ab | 321327 | CP000239 | 0 | *Cylindrospermopsis raciborskii* CS-505 | 533240 | ACYA01000000 | 1 |
| *Synechococcus* sp. WH 8102 | 84588 | BX548020 | 0 | *Cylindrospermum stagnale* PCC 7417 | 56107 | CP003642 | 1 |
| *Thermosynechococcus elongatus* BP-1 | 197221 | BA000039 | 0 | *Nodularia spumigena* CCY9414 | 313624 | AAVW01000000 | 1 |
| **Subsection II** |  |  |  | *'Nostoc azollae'* 0708 | 551115 | CP002059 | 1 |
| *Chroococcidiopsis thermalis* PCC 7203 | 251229 | CP003597 | 0 | *Nostoc* sp. PCC 7120 | 103690 | BA000019 | 1 |
| *Chroococcidiopsi*s sp. PCC 8201 | this study |  | 0 | *Microchaete* sp. PCC 7126 | 643473 | ANFJ01000000 | 1 |
| *Stanieria cyanosphaera* PCC 7437 | 111780 | CP003653 | 0 | **Subsection V** |  |  |  |
| *Xenococcus* sp. PCC 7305 | 102125 | ALVZ01000000 | 0 | *Chlorogloeopsis fritschii* PCC 6912 | 211165 | AJLN01000000 | 1 |
| **Subsection III** |  |  |  | *Chlorogloeopsis fritschii* PCC 9212 | 184925 | AJLM01000000 | 1 |
| *Arthrospira platensis* NIES-39 | 696747 | AP011615 | 1 | *Fischerella thermalis* PCC 7521 | 98439 | AJLL01000000 | 1 |
| *Arthrospira maxima* CS-328 | 513049 | ABYK01000000 | 1 | *Fischerella muscicola* PCC 7414 | 306281 | AJLK00000000 | 1 |
| *Coleofasciculus chthonoplastes* PCC 7420 | 118168 | ABRS01000000 | 1 | *Fischerella* sp. PCC 9339 | 1174528 | ALVS01000000 | 1 |
| *Crinalium epipsammum* PCC 9333 | 1173022 | CP003620 | 1 | *Mastigocladopsis repens* PCC 10914 | 221288 | ALVW00000000 | 1 |
| *Geitlerinema* sp. PCC 7407 | 1173025 | CP003591 | 1 |  |  |  |  |
