# Supplementary material for: Cyanobacteria and the Great Oxidation Event: evidence from genes and fossils
Source: Palaeontology. 2015 Jun 23;58(5):769–85. doi: 10.1111/pala.12178 (PMC4755140; doi:10.1111/pala.12178)
Supplement: Supplementary file 6 — Table S2. Prior transition rates for the character state reconstruction applying Bayesian inference. [file PALA-58-769-s006.docx]

|  | **q01** | **q10** | **Harmonic mean** |
| --- | --- | --- | --- |
| Analysis 0 | 0-100 | 0-100 | -35.450319 |
| Analysis 1 | 0-1.2 | 0.5-1.7 | -28.570444 |
| Analysis 2 | 0.1-0.9 | 0.7-1.5 | -28.232523 |
| Analysis 3 | 0.3-0.7 | 0.9-1.3 | -27.966509 |
|  |  |  |  |
