# Supplementary material for: Cyanobacteria and the Great Oxidation Event: evidence from genes and fossils
Source: Palaeontology. 2015 Jun 23;58(5):769–85. doi: 10.1111/pala.12178 (PMC4755140; doi:10.1111/pala.12178)
Supplement: Supplementary file 7 — Table S3. Reconstructed ancestral character states. [file PALA-58-769-s007.docx]

|  | **Maximum Likelihood analyses** | | | |
| --- | --- | --- | --- | --- |
| **Node** | **asymmetrical rates** | | **equal rates** | |
|  | **unicellular** | **multicellular** | **unicellular** | **multicellular** |
| **66** | 0.38396422 | 0.61603578 | 0.487776323 | 0.512223677 |
| **67** | 0.254411063 | 0.745588937 | 0.387102937 | 0.612897063 |
| **68** | 0.993811806 | 0.006188194 | 0.994766507 | 0.005233493 |
| **69** | 0.11765938 | 0.88234062 | 0.261251722 | 0.738748278 |
| **70** | 0.0497144 | 0.9502856 | 0.134302696 | 0.865697304 |
| **71** | 0.055185089 | 0.944814911 | 0.136506198 | 0.863493802 |
| **72** | 0.016076914 | 0.983923086 | 0.053225513 | 0.946774487 |
| **73** | 0.111305494 | 0.888694506 | 0.253972155 | 0.746027845 |
| **74** | 0.384065826 | 0.615934174 | 0.482839813 | 0.517160187 |
| **75** | 0.999486562 | 0.000513438 | 0.999598622 | 0.000401378 |
| **76** | 0.079156987 | 0.920843013 | 0.208530139 | 0.791469861 |
| **77** | 0.175457207 | 0.824542793 | 0.306668022 | 0.693331978 |
| **78** | 0.52340158 | 0.47659842 | 0.607724188 | 0.392275812 |
| **79** | 0.987150964 | 0.012849036 | 0.99092841 | 0.00907159 |
| **80** | 0.999094706 | 0.000905294 | 0.9995227 | 0.0004773 |
| **81** | 0.999911598 | 8.84E-05 | 0.999944063 | 5.59E-05 |
| **82** | 0.00665343 | 0.99334657 | 0.035264374 | 0.964735626 |
| **83** | 0.008255323 | 0.991744677 | 0.02936679 | 0.97063321 |
| **84** | 0.000867996 | 0.999132004 | 0.009397537 | 0.990602463 |
| **85** | 0.003820166 | 0.996179834 | 0.011335775 | 0.988664225 |
| **86** | 0.000255156 | 0.999744844 | 0.0036751 | 0.9963249 |
| **87** | 0.000624588 | 0.999375412 | 0.001720235 | 0.998279765 |
| **88** | 0.000115533 | 0.999884467 | 0.00017568 | 0.99982432 |
| **89** | 0.000707415 | 0.999292585 | 0.00154804 | 0.99845196 |
| **90** | 0.001580177 | 0.998419823 | 0.002619139 | 0.997380861 |
| **91** | 4.31E-05 | 0.999956912 | 6.83E-05 | 0.999931724 |
| **92** | 0.001321623 | 0.998678377 | 0.006245817 | 0.993754183 |
| **93** | 0.004799694 | 0.995200306 | 0.009699332 | 0.990300668 |
| **94** | 0.219469994 | 0.780530006 | 0.262695264 | 0.737304736 |
| **95** | 0.996930304 | 0.003069696 | 0.997499614 | 0.002500386 |
| **96** | 5.03E-05 | 0.999949657 | 0.000144723 | 0.999855277 |
| **97** | 0.000200243 | 0.999799757 | 0.000341113 | 0.999658887 |
| **98** | 0.000300009 | 0.999699991 | 0.000474882 | 0.999525118 |
| **99** | 5.05E-08 | 0.999999949 | 7.98E-08 | 0.99999992 |
| **100** | 6.04E-05 | 0.999939579 | 9.87E-05 | 0.999901253 |
| **101** | 9.85E-06 | 0.999990149 | 1.63E-05 | 0.999983723 |
| **102** | 6.45E-05 | 0.999935507 | 0.000128864 | 0.999871136 |
| **103** | 9.40E-06 | 0.999990598 | 1.83E-05 | 0.99998172 |
| **104** | 0.000198486 | 0.999801514 | 0.000304111 | 0.999695889 |
| **105** | 3.27E-05 | 0.999967335 | 5.09E-05 | 0.999949091 |
| **106** | 2.56E-05 | 0.999974411 | 4.32E-05 | 0.999956814 |
| **107** | 6.06E-05 | 0.999939357 | 9.76E-05 | 0.999902418 |
| **108** | 3.52E-05 | 0.999964816 | 6.02E-05 | 0.999939813 |
| **109** | 5.49E-05 | 0.999945074 | 9.20E-05 | 0.999908021 |
| **110** | 0.010099589 | 0.989900411 | 0.0219132 | 0.9780868 |
| **111** | 0.054087389 | 0.945912611 | 0.073119519 | 0.926880481 |
| **112** | 0.027438673 | 0.972561327 | 0.046484732 | 0.953515268 |
| **113** | 0.001656639 | 0.998343361 | 0.004787957 | 0.995212043 |
| **114** | 0.000545379 | 0.999454621 | 0.002087845 | 0.997912155 |
| **115** | 0.000837101 | 0.999162899 | 0.00203848 | 0.99796152 |
| **116** | 0.643735052 | 0.356264948 | 0.709197454 | 0.290802546 |
| **117** | 0.636883555 | 0.363116445 | 0.705037883 | 0.294962117 |
| **118** | 0.989075132 | 0.010924868 | 0.992781451 | 0.007218549 |
| **119** | 0.990885424 | 0.009114576 | 0.994686379 | 0.005313621 |
| **120** | 0.99386885 | 0.00613115 | 0.996797094 | 0.003202906 |
| **121** | 0.996663019 | 0.003336981 | 0.997696155 | 0.002303845 |
| **122** | 0.998462249 | 0.001537751 | 0.999449818 | 0.000550182 |
| **123** | 0.999616287 | 0.000383713 | 0.999818172 | 0.000181828 |
| **124** | 0.9991942 | 0.0008058 | 0.999242875 | 0.000757125 |
| **125** | 0.999010239 | 0.000989761 | 0.998938017 | 0.001061983 |
| **126** | 0.99846297 | 0.00153703 | 0.999231279 | 0.000768721 |
| **127** | 0.996831321 | 0.003168679 | 0.99770647 | 0.00229353 |
| **128** | 0.891038327 | 0.108961673 | 0.896297764 | 0.103702236 |
| **129** | 0.998974221 | 0.001025779 | 0.999069029 | 0.000930971 |
