# Supplementary material for: Cyanobacteria and the Great Oxidation Event: evidence from genes and fossils
Source: Palaeontology. 2015 Jun 23;58(5):769–85. doi: 10.1111/pala.12178 (PMC4755140; doi:10.1111/pala.12178)
Supplement: Supplementary file 8 [file PALA-58-769-s008.docx]

**Figure S1.** Maximum Likelihood tree of 65 cyanobacterial taxa based on concatenated 16S and 23S ribosomal RNA sequences (4631 nucleotide sites). Maximum Likelihood bootstrap support is shown on branches. Cyanobacterial taxa are colour coded. Unicellular taxa belonging to morphological subsections I and II are displayed in yellow and orange, whereas multicellular cyanobacterial taxa belonging to subsections III, IV and V are shown in green, blue and pink. Many deep divergence events (nodes) are only weakly supported.

**Figure S2.** Tree is based on 756 proteins of 65 cyanobacterial taxa. Displayed is an ultrametric tree with node numbers as presented in Table S3.

**Figure S3.** (a) Transition rates from uni- to multicellularity (q01) and back (q10) were estimated during four different MCMC searches. The rates were samples from uniform prior distributions of varying ranges (see Table S2). (b) Estimated Likelihoods and (c) posterior distributions of character states at node 69. Choosing a narrower prior distribution towards values observed in the ML estimates (q01: 0.576; q10: 0.8254) increased Likelihood estimations and posterior support for a multicellular character state at node 69.

**Figure S4.** (a) Divergence time estimation without calibration 2 and (b) effective prior ages (red) and posterior age estimates (black) for the origin of cyanobacteria (calibration 1), the origin of section IV and V cyanobacteria (calibration 2) and the origin of multicellularity (calibration 3). The origin of multicellularity is estimated to have occurred before the GOE.

**Table S1.** Strains with taxa ID and accession numbers from GenBank, and character states (multi- or unicellular). Cyanobacteria have been grouped into five subsections. Where section I and II comprise unicellular taxa and section III to V multicellular taxa. Colour code displayed in the table matches color code used for the section in the other figures.

**Table S2.** Uniform prior distributions from which transition rates for the character state reconstruction are sampled during the MCMC analysis. For each prior distribution the final (after the MCMC) harmonic mean as calculated by the software is presented. Narrowing the uniform distribution increases the harmonic mean.

**Table S3.** Ancestral character states for the cyanobacterial phylum applying maximum likelihood analyses with asymmetrical and equal transition rates. Node numbers refer to nodes illustrated in Figure S3.
